# Influence of Age, Sex and Calendar Year on Lifetime Accumulated Red Bone Marrow Dose from Diagnostic Radiation Exposure

**DOI:** 10.1371/journal.pone.0078027

**Published:** 2013-11-11

**Authors:** Wolfgang Hoffmann, Merle Friederike Meiboom, Kerstin Weitmann, Claudia Terschüren, Heiner von Boetticher

**Affiliations:** 1 Institute for Community Medicine, Department Epidemiology of Health Care and Community Health, University Medicine Greifswald, Greifswald, Germany; 2 Institute for Radiology, Hospital Links der Weser, Bremen, Germany; 3 Institute for Radiology and Academy of Radiation Protection, Hospital Links der Weser, Bremen, Germany; Northwestern University Feinberg School of Medicine, United States of America

## Abstract

Our aim is to evaluate the relevance of different factors influencing lifetime accumulated red bone marrow dose, such as calendar year, age and sex. The lifetime dose was estimated for controls interviewed in person (N = 2811, 37.5% women) of the population-based representative Northern Germany Leukemia and Lymphoma Study. Data were assessed in standardized computer-assisted personal interviews. The calculation of doses is based on a comprehensive quantification model including calendar year, sex, kind of examination, and technical development. In multivariate regression models the annual red bone marrow dose was analyzed depending on age, sex and calendar year to consider simultaneously temporal changes in radiologic practice and individual risk factors. While the number of examinations continuously rises over time, the dose shows two peaks around 1950 and after 1980. Men are exposed to higher doses than woman. Until 1970 traditional examinations like conventional and mass screening examinations caused the main dose. They were then replaced by technically advanced examinations mainly computed tomography and cardiac catheter. The distribution of the red bone marrow dose over lifetime depends highly on the technical standards and radiation protection survey. To a lesser extent it is influenced by age and sex of the subjects. Thus epidemiological studies concerning the assessment of radiation exposure should consider the calendar year in which the examination was conducted.

## Introduction

Radiation is a risk factor for malignant diseases such as leukemia, malignant lymphoma, and solid tumors but also for benign medical conditions like thyroid nodules, eye cataract and impaired brain development [1]–[6]. Compared to other diseases caused by radiation exposure leukemia develops more frequently also after lower doses and with shorter latency following exposure. Latency ranges between 2–25 years with a peak at 7–8 years [7]. Leukemia is one of the best studied radiation induced diseases. It has its origin in the red bone marrow, which is characterized by a distribution over multiple parts of the body (head, extremities, thorax and abdomen) and a certain shielding by the surrounding bone. Depending on exposure conditions, red bone marrow dose may differ significantly from the effective dose. The effective dose of an examination represents the uniform whole-body dose which results in the same radiation risk as the organ doses absorbed [8].

To quantify medical radiation dose it is evident to consider a variety of factors, so for example the date of an examination is crucial as the technical development has led to a reduction of doses over time.

Unfortunately, there is no generally accepted methodology for the quantification of medical radiation dose. Published models differ significantly. They range from simple models, based only on the number of x-ray examinations [5], [9]–[11] occasionally augmented by factors for the type of examination [5], to more complex quantifications considering technical developments over time, real life conditions and the accumulated dose to specific critical organs [12].

Therefore it is of great importance, to choose a comprehensive model that adequately considers all relevant factors that might influence patient dose, like calendar year, state of technical improvement, radiological practice and number and spectrum of conventional and advanced examinations. Our aim is to evaluate the relevance of different factors influencing the lifetime accumulated red bone marrow dose in a general population.

## Materials and Methods

### Ethics Statement

The study design of the Northern Germany Leukemia and Lymphoma Study (NLL) was approved by the ethics committee and endorsed by the Medical Associations and the Associations of Statutory Health Insurance Physicians of the Federal States of Lower Saxony, Schleswig-Holstine and Hamburg.

### Northern Germany Leukemia and Lymphoma Study

This study is based on the Northern Germany Leukemia and Lymphoma Study (NLL), a large population-based epidemiologic case-control study on causes and risk factors for monoclonal malignant hematologic diseases [13]. All incident cases at ages younger than 75 years between 1986 and 1998 in six counties of Northern Germany were included. For each case at least two controls were recruited at random from population registries and matched by age, gender and region.

All analyses for this study were restricted to the controls of the NLL, who were randomly sampled from 78 population registries covering the population in the study area (approximate 1.1 million). Leukemia and lymphoma cases were excluded to avoid systematic selection and any possible bias due to additional examinations during patients' diagnostic workup and staging of the disease [14]. All controls included in this study were interviewed in person. Interviews with family members were excluded considering that family members may underestimate the number of examinations the index person really had - a problem that had been shown previously for the use of household appliances ]14]. Altogether this study is based on 2811 interviews (Table 1). Out of this sample three birth cohorts (1920–1929, 1940–1949, 1960–1969) were selected (total N = 1458) which represent three different generations for comparison (Table 1).

**Table 1:** Age distribution of the population based sample in this study including all control subjects of the NLL interviewed in person.

Birth year		Men	Women	Total	
1910–1914		25	11	36	
1915–1919		74	36	100	
**1920–1924**	***cohort 1***	**225**	**126**	**351**	***748***
**1925–1929**		**253**	**144**	**397**	
1930–1934		266	188	454	
1935–1939		248	137	385	
**1940–1944**	***cohort 2***	**216**	**152**	**368**	***558***
**1945–1949**		**116**	**74**	**190**	
1950–1954		104	67	171	
1955–1959		54	31	85	
**1960–1964**	***cohort 3***	**36**	**30**	**66**	***152***
**1965–1969**		**61**	**25**	**86**	
1970–1974		32	12	44	
1975–1979		14	7	21	
1980–1984		26	10	36	
1985–1989		6	5	11	
**1910–1989**		**1756**	**1055**	**2811**	**1458**

Three birth cohorts (1920–1929, 1940–1949 and 1960–1969) (highlighted) are selected to represent three different generations.

Standardized, personal computer-assisted face-to-face interviews were conducted by specially trained interviewers. Six kinds of radiologic examinations were addressed in separate sections of the interview: mass screening examinations (to screen for tuberculosis), computed tomography (CT), cardiac catheter examinations including interventions, examinations with contrast medium, conventional examinations, and nuclear medical examinations. The groups were defined based on differences in the used equipment, preparation of the patients, and/or the departments involved. Short introductory explanations should trigger subjects' pertinent memories. Thus, for example, mass screening examinations of the thorax especially for early diagnosis of tuberculosis were embedded in a special organization that distinguishes them from conventional examinations in a hospital or a radiological practice. Only at least five examinations over one's lifetime in the screening program were considered as mass screening examinations. Less frequent screening examinations were categorized to the conventional examinations in the analyses.

CT examinations can be mistaken for magnetic resonance imaging. To eliminate this potential misclassification the subjects were informed about characteristic differences between these types of examinations using pictures cards and a short text addressing the characteristics from the point of view of a patient (e.g. low humming, compulsory taking off the watch, use of lead apron).

For each examination the kind, exposed body area, calendar year, and institution as well as age and sex of the subject were assessed.

### Model conception

There is a large variation in patient dose for any given x-ray examination which can vary by 1 or 2 orders of magnitude depending on the equipment used and a lot of dose-modifying factors which correspond to higher-than-necessary patient dose in real lifetime radiological practice. The advancement in radiologic technique has significantly reduced patient dose over time for many x-ray applications. This study needs to account for examinations carried out over the time of the study. To deduce an “average dose value” would not take into account the advancement of radiological technology. Another problem is that published dose values are often obtained in vitro under ideal experimental conditions and therefore underestimate dose values for real patients. The variability of quality standards in radiologic institutes is changing with time too caused by an increasing concern for radiation exposure from medical sources. So the standard of radiologic practice in the prevailing period has to be taken into account.

Von Boetticher and Hoffmann applied a comprehensive model for retrospective dose assessment to determine lifetime radiation exposure to patients [13]. This model is based on the knowledge that for a given examination at a certain time the dose under ideal conditions is a relative exact quantity. The model includes two sets of correction factors, one for the time of the examination (state of technology) and another one for the assumed quality standard.

For this model from a multitude of historic sources a set of correction factors for the state of radiologic technology was derived: For example since the 1940's, the development of the film screen imaging system allowed for a speed improvement of approximately a factor of 2 in each consecutive decade. For x-ray fluoroscopic examinations the most important technical advancement was the replacement of the passive screen through the image-amplifier-TV technique, reducing the previous screen dose by a factor of 4–5. In population-based chest screening the introduction of image-amplifier-TV systems was accompanied by a dose reduction by a factor of 20. For CT in the model no correction factors for technical advancement are provided, because in the relevant time period a reduction of patient dose was not achieved.

The second set of correction factors considers the prevailing standard of radiologic practice with respect to patient dose (ideal standard A: correction factor for patient dose of 1; lower realistic standard B: factor of 2; medium realistic standard C: factor of 4; upper realistic standard D: factor of 8). The optimum conditions are usually restricted to experimental settings with dose measurements under lab conditions based on anthropometric phantoms and can rarely if ever be met in real life radiologic practice.

We used the matrix published in [12] of the combined two sets of correction factors for the quantification of relevant red bone marrow doses in the studied time period from conventional x-ray examinations, conventional fluoroscopy with contrast media, chest x-ray population screening and cardiac catheterization with and without intervention. The factors are standardized on doses for the period 1976–1985 under ideal conditions (standard A of radiologic practice).

In this model all published doses to the red bone marrow related to a time period and a defined diagnostic standard may be used as basic data. In this study we use a set of ideal doses measured under optimum conditions referring to the period of 1976–1985 published in [12] too. For diagnostic nuclear medicine average patient doses also provided in [12] were applied.

### Data Analysis

Analysis was performed with SAS (Statistical Analysis System, Version 9.1, SAS Institute, Cary, NC (USA)). Main analyses were restricted to three birth cohorts (1920–1929, 1940–1949, 1960–1969; N = 1458) from the controls of the NLL. Descriptive statistics as mean, standard deviation (STD) and range are displayed.

Excel 2007 (Microsoft, Redmond, USA) was used to create graphs. Unless mentioned otherwise, all figures display three years moving averages of the respective annual patient doses or number of examinations. Moving averages are used to allow for some smoothing of the random influencing of single calendar year data.

We conducted linear regression models with the annual red bone marrow dose per calendar year and person as dependent variable and age and calendar year as predictors for each birth cohort, respectively and for all persons together (N = 2811) adjusting for sex. An interaction term age*calendar year was included in all models.

## Results

### Number of examinations

**Figure 1:**
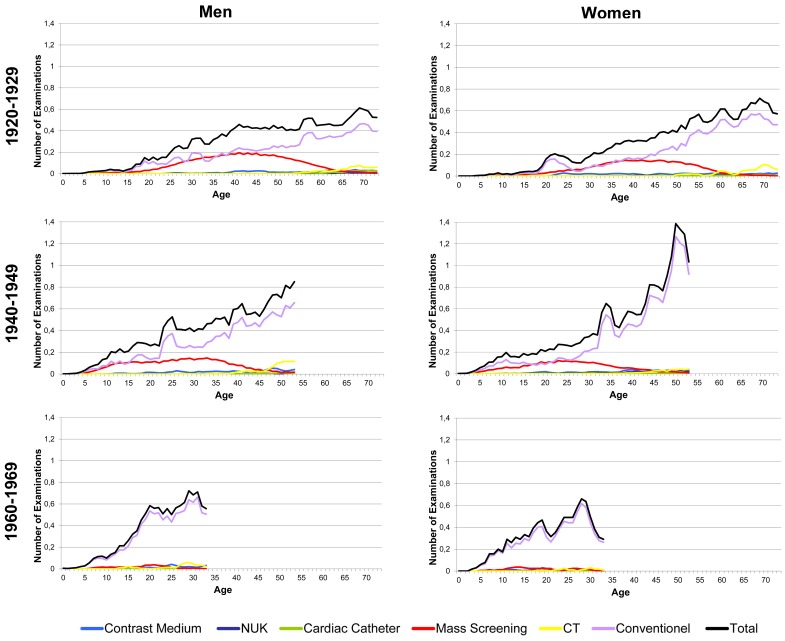
Average number of the different kinds of examinations (3 years moving averages) considering all three birth cohorts separately for male and female subjects. The number of examinations rises continuously over lifetime. This rise with age has become significantly steeper in more recent birth cohorts. (NUK: nuclear medical examinations, CT: computed tomography).

The number of radiologic examinations for diagnostic purposes increases continuously with age in all cohorts. The rise appears to be less steep in childhood and young adult age and becomes steeper in later life (Figure 1).

For a given age, the number of examinations rises over consecutive cohorts and is highest in the most recent cohort. Comparing the birth cohorts 1920–1929 and 1940–1949, a 40 year old male in the more recent cohort accumulates on average twice the number of examinations over life than a male of the same age in the earlier cohort (6.3 (STD: 10.7) vs. 12.1 (STD: 13.4) average cumulative number of examinations) (data not shown). A similar trend can be observed for females (5 (STD: 12.7) vs. 10.4 (10.4) cumulative number of examinations). Subjects in the birth cohort 1960–1969 (e.g. at an age of 30 years) had on average 3.5 to 4 times more examinations than a 30 year old subject in the birth cohort 1920–1929 (male: 9.7 (STD: 8.3) vs. 2.8 (STD: 6.3); female: 9.3 (STD: 8.8) vs. 2.3 (STD: 10.9)).

**Figure 2:**
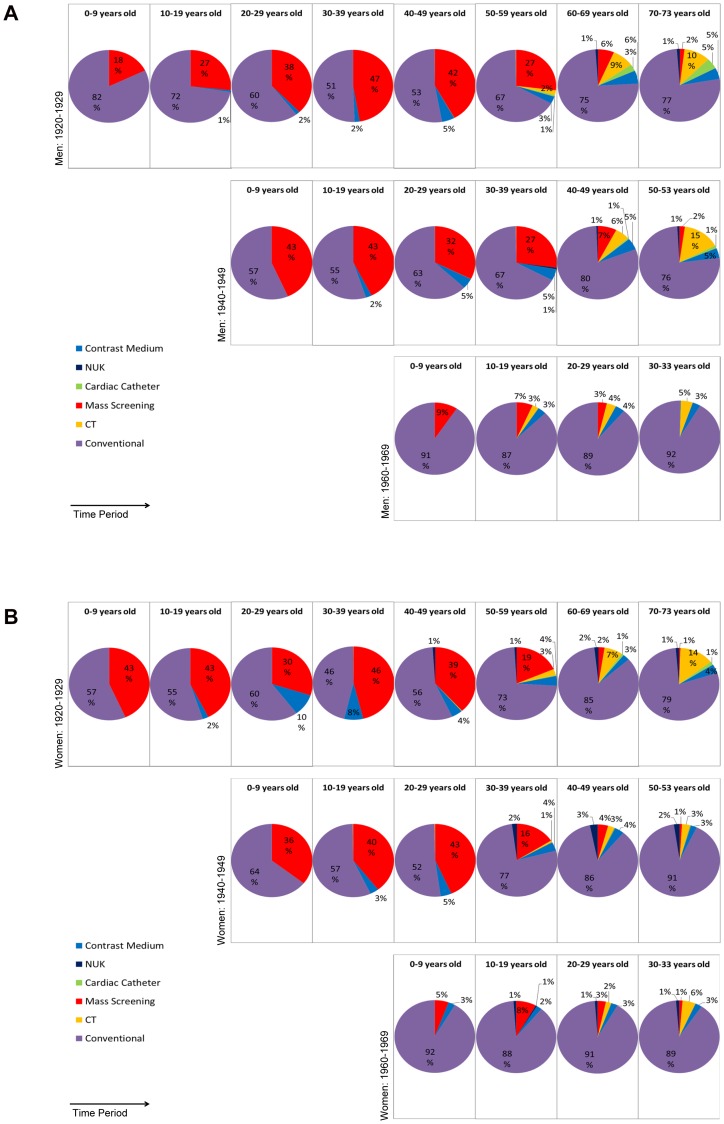
Distribution of the number of different kinds of examinations considering all three birth cohorts separately for male and female subjects. The diagrams of the birth cohorts show a similar distribution when compared after an age shift of twenty years in such a way that the same time period is listed below one another. It can be noted that the number is generated mainly by conventional examinations. In the older subjects mass screening examinations are responsible for a considerable proportion of all examinations. **Figure 2a** **:** Men. **Figure 2b** **:** Women. (NUK: nuclear medical examinations, CT: computed tomography).

Between all birth cohorts the proportions of the different kinds of examination change (Figure 2). Conventional radiological examinations account for the largest proportion of examinations over all birth cohorts. Depending on the year of birth and the age this fraction ranges from 46–92%. The difference is mainly caused by mass screening that was more prevalent in the early birth cohorts, particularly in young years. Over lifetime, the number of mass screening examinations accounts for 26.6% of all radiologic examinations for men born between 1920–1929 and for at least 20% for men born between 1940–1949 (data not shown). This proportion is smaller in women (1920–1929 cohort: 19%; 1940–1949 cohort: 14%). Over time the number of mass screening examinations has decreased (Figure 1, 2). Subjects born after 1970 no longer received mass screening examinations.

In terms of the number of examinations, the contribution of CT and cardiac catheter is small. Subjects of the birth cohorts 1920–1929 and 1940–1949 accumulate more examinations (on average 1.1 and 1.0, respectively, per male subject over life) than subjects of the birth cohort 1960–1969 (on average 0.4 per male subject over life) (data not shown). Especially cardiac catheter examinations are applied to older subjects more often than to younger subjects. However, subjects in the most recent birth cohort of 1960–1969 were exposed to these examinations earlier in life than were subjects in the birth cohort of 1920–1929. The data for women follow a similar trend to those of men, although women generally seem to be less exposed to these examinations, especially to cardiac catheter examinations (Figure 2). Nuclear medical examinations contribute only a minor fraction to the overall number of examinations (Figure 2).

The distributions of the different kinds of examination become more similar when the cohorts are compared at periods of time rather than at age (Figure 2). Thus, the distributions appear to depend more on calendar year than on age.

### Dose to the red bone marrow

**Figure 3:**
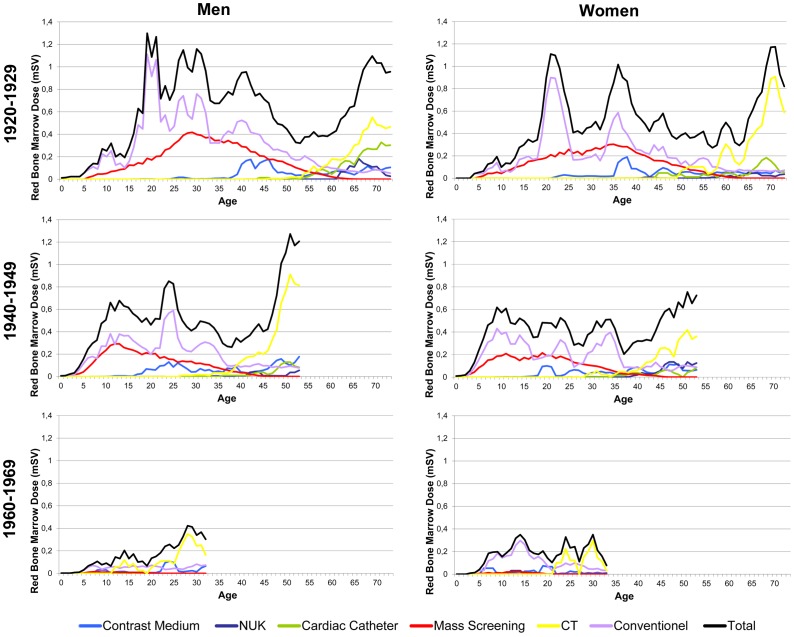
Average red bone marrow dose per age (3 years moving averages) for the different birth cohorts separated by gender. A drop after an increase accompanied by several peaks and a second rise in later years can be observed in the birth cohorts 1920–1929 and 1940–1949. While the first increase is generated by conventional kinds of examinations, the second rise is caused by technically advanced examinations. (NUK: nuclear medical examinations, CT: computed tomography).

The annual dose to the red bone marrow shows two peaks over lifetime in the cohorts 1920–1929 and 1940–1949 (Figure 3).

**Figure 4:**
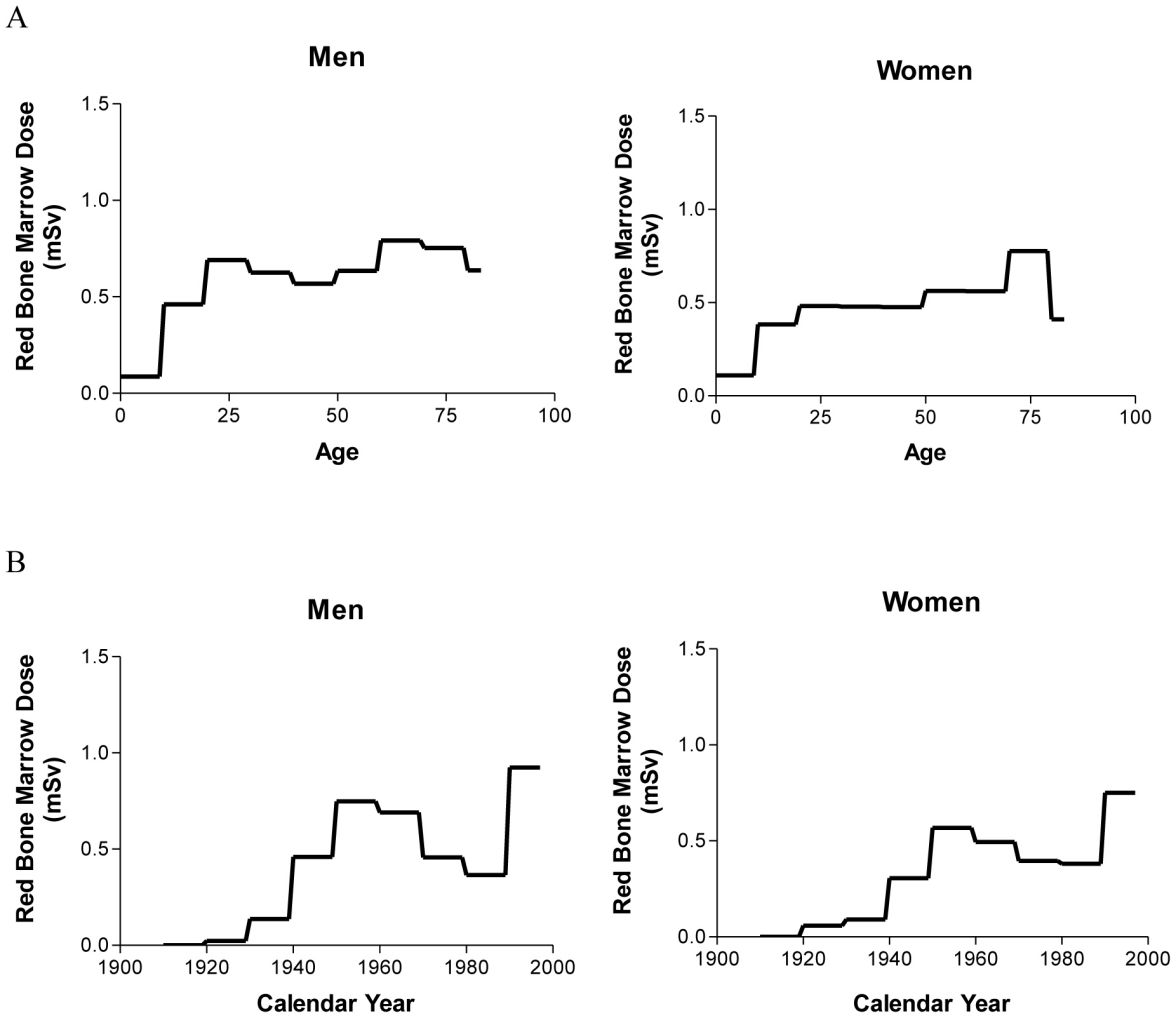
Red bone marrow dose in association with age, calendar year and gender. **A:** Red bone marrow dose per age and gender considering all subjects of the sample (N = 2811) independent of birth year. After a rise of dose in childhood the dose per calendar year shows no significant alteration until old age. **B:** Red bone marrow dose per calendar year considering all subjects of the sample independent of birth year and just separated by gender. A bimodal distribution of dose with an early rise followed by a drop and a second increase can be noticed. **A + B** In both graphics the dose were averaged over 10 years for smoothing random peaks.

In both cohorts the first rise is accompanied by several peaks and is followed by a decrease. Both are not matched with age. While the minimum of dose in the birth cohort of 1920–1929 is around the age of 55–60, the minimal dose in the birth cohort of 1940–1949 is around the age of 35–40. Comparing the difference between the time of birth of both cohorts and the difference of age at the dose-minimum, both appear to be in the same range of around twenty years. In Figure 4 the dose of all subjects (independent of birth year and only categorized by gender) is plotted against age and calendar year. The plot indicates that the ups and downs depend on the calendar year rather than on the subjects' age. In fact, the dose does not change much between an age of 25–70 over all subjects in the sample.

The first rise in patient dose is generated nearly exclusively by traditional examinations like conventional examinations and mass screening examinations (Figure 3).

**Figure 5:**
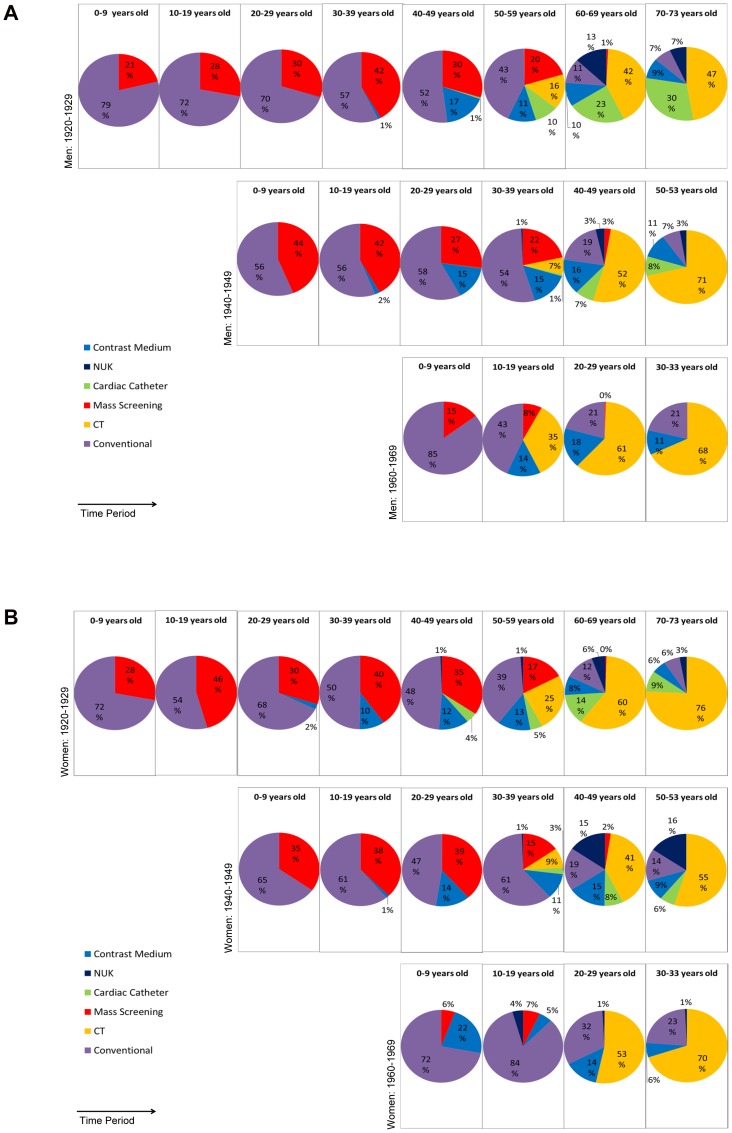
Distribution of the red bone marrow dose generated by the different categories of examinations for the three birth cohorts for male and female subjects. The distribution changed from a predominance of conventional and mass screening examinations to a dose generated mainly by technically advanced examinations like computed tomography and cardiac catheter examinations but also nuclear medicine and contrast medium examinations. The vertical dimension formation reflects similar calendar time. The diagrams of the birth cohorts show a similar distribution when compared at a given calendar time. **Figure 5a** **:** Men. **Figure 5b** **:** Women. (NUK: nuclear medical examinations, CT: computed tomography).

The second rise depends on advanced kinds of examinations including CT, cardiac catheter and other examinations with contrast medium and fluoroscopy as well as nuclear examinations (Figure 3). Here conventional examinations play only a minor role while CT examinations cause the major proportion of total dose. Comparing the birth cohorts 1920–1929 and 1940–1949 the second rise differs with respect to the fraction of CT and cardiac catheter examinations. Men born between 1920 and 1929 were exposed to cardiac catheter on average more often and therefore accumulated a considerable dose from this kind of examination (up to 30% of the overall dose per year; Figure 5). In contrast, the birth cohort of 1940–1949 received lower doses from cardiac catheter examinations but acquired higher doses from CT scans. Women in the birth cohort 1920–1929 were exposed to less cardiac catheter examinations but to more CT examinations compared to men (Figure 3).

The most recent birth cohort (1960–1969) does not show the first rise and fall observed in the older birth cohorts. However, the transition from doses generated by conventional examinations to the dose caused by CT and examinations with contrast medium is as considerable as it is in the other birth cohorts. Although the dose caused by conventional examinations still causes a relevant proportion of lifetime accumulated dose in this birth cohort, CT examinations generate the major part of the overall dose in adults. In accordance with the age distribution for cardiac catheter examinations in the other cohorts subjects born between 1960 and 1969 achieve no relevant dose by catheter examinations over the time of their follow up (Figure 5).

Contrary to the number of examinations, the overall dose generally appears to decrease from cohort 1920–1929 to 1940–1949 and to 1960–1969 (Table 2).

**Table 2:** Red bone marrow dose accumulated until the mentioned age and its proportion of the reference value (total dose of the cohort 1920–1929 at the age of 70).

Men	Accumulated red bone marrow dose (mSv)			Fraction			
Age	1920–1929	1940–1949	1960–1969	Age	1920–1929	1940–1949	1960–1969
≤5	0.12	0.28	0.03	≤5	0%	1%	0%
≤10	1.22	2.27	0.43	≤10	3%	5%	1%
≤15	2.19	5.38	0.96	≤15	5%	13%	2%
≤20	7.09	7.97	1.61	≤20	17%	19%	4%
≤25	10.35	11.44	2.66	≤25	24%	27%	6%
≤30	15.87	13.73	4.16	≤30	37%	32%	10%
≤35	19.96	16.12		≤35	47%	38%	
≤40	24.17	17.57		≤40	57%	41%	
≤45	28.18	19.53		≤45	66%	46%	
≤50	30.84	23.41		≤50	73%	55%	
≤55	32.59			≤55	77%		
≤60	34.52			≤60	81%		
≤65	37.33			≤65	88%		
≤70	42.42			≤70	100%		

Women	Accumulated red bone marrow dose (mSv)			Fraction			
Age	1920–1929	1940–1949	1960–1969	Age	1920–1929	1940–1949	1960–1969
≤5	0.12	0.43	0.04	≤5	0%	1%	0%
≤10	0.77	3.06	0.90	≤10	2%	9%	3%
≤15	1.80	5.63	2.32	≤15	6%	17%	7%
≤20	4.04	7.72	3.26	≤20	12%	24%	10%
≤25	8.74	9.98	4.31	≤25	27%	31%	13%
≤30	10.97	11.68	5.37	≤30	34%	36%	16%
≤35	14.10	14.11		≤35	43%	43%	
≤40	18.06	15.45		≤40	55%	47%	
≤45	20.53	17.23		≤45	63%	53%	
≤50	22.56	20.39		≤50	69%	62%	
≤55	24.60			≤55	75%		
≤60	26.39			≤60	81%		
≤65	28.13			≤65	86%		
≤70	32.63			≤70	100%		

The fraction decreases with rising birth year. The only exceptions are subjects of the birth cohorts 1940–1949 in childhood and as young adults. These groups are exposed to higher doses than the previous birth cohort 1920–1929.

While women in the earlier cohorts accumulated less radiation dose than men, this sex difference decreases over time and is no longer present in the more recent cohort.

**Figure 6:**
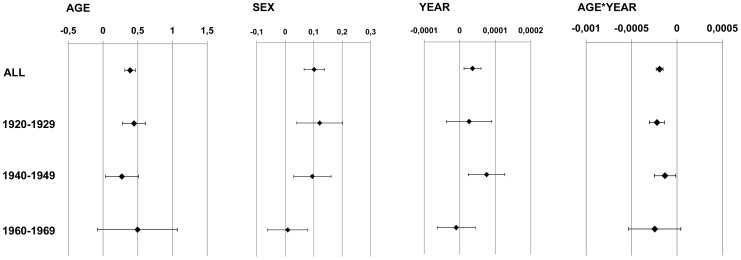
Point estimates and 95% confidence limits of the multivariate regression model.

**Figure 7:**
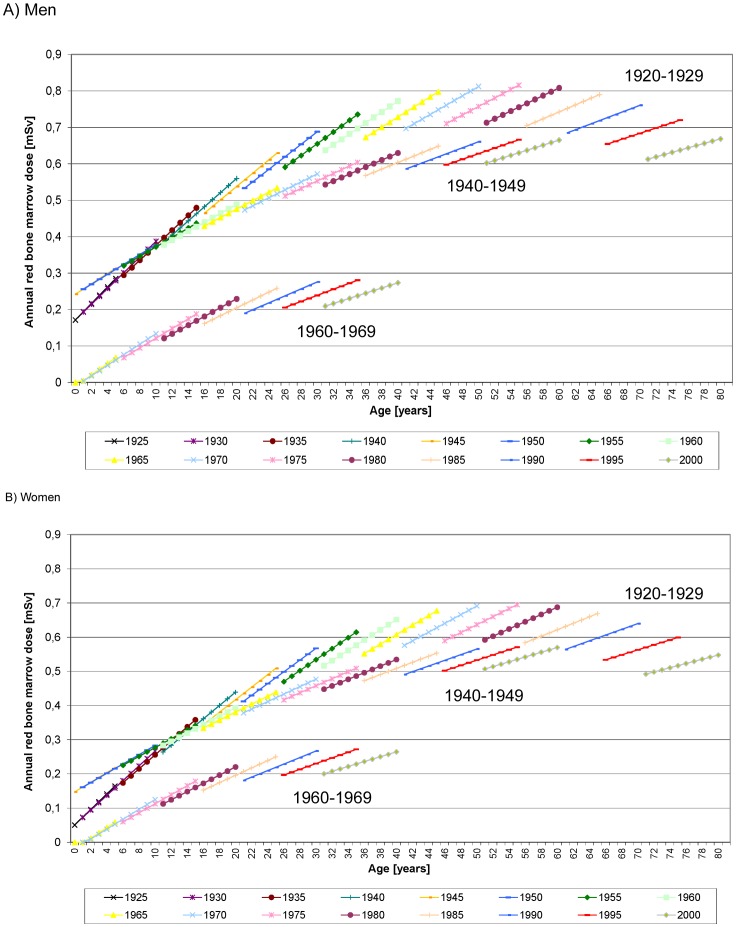
Annual red bone marrow dose by age and calendar year for the three birth cohorts for A) Men, B) Women) based on the multivariate regression model (Table 3). Each line represents an age range of ten years, determined by the definition of the respective cohorts. Colours indicate calendar years restricted to 5-years intervals starting with 1925. All lines show an increase of annual dose with ten consecutive birth cohorts. Adjacent lines depict annual doses of the same birth cohorts for later calendar years in five year steps. This allows a comparison between the impact of increasing age and secular trend over calendar years. The increase of annual dose is faster over younger years and levels off later in life. The dynamics are basically similar over the three subsequent birth cohorts. The annual dose however with respect to age has considerably decreased over calendar time.

In order to differentiate between the influence of age in the three cohorts from the strong influence of the calendar year multivariate regression models (Table 3, Figure 6) are conducted. They show an age-dependent dose increase (1920–1929, *P*<0.0001; 1940–1949: *P* = 0.02; 1960–1969: *P* = 0.09), which levels off for older ages. In Figure 7 the dynamic of the red bone marrow dose per year is shown for these three variables separately for the three birth cohorts. The initial increase is stronger in earlier birth cohorts.

**Table 3:** Linear regression models of the annual red bone marrow dose by sex, age and calendar year for all persons together and the three birth cohorts (CI: confidence interval).

	ß-coefficient	CI	STD-error	*P* value
**all (183395 years of 2811 persons) R^2^ = 0.02, P<0.001**				
sex (1:male, 0: female)	0.10174	(0.06599;0.13749)	0.01824	<0.0001
age	0.38939	(0.31327;0.46550)	0.03884	<0.0001
calendar year	0.00003597	(0.00001204;0.0000599)	0.00001221	0.0032
age*calendar year	−0.00019125	(−0.00022933;−0.00015316)	0.00001943	<0.0001
**1920–1929 (58587 years of 748 persons) R^2^ = 0.02, P<0.001**				
sex (1:male, 0: female)	0.12078	(0.04055;0.20100)	0.04093	0.0032
age	0.44655	(0.28088;0.61222)	0.08452	<0.0001
calendar year	0.00002631	(−0.00003741;0.00009003)	0.00003251	0.4184
age*calendar year	−0.00022018	(−0.00030291;−0.00013744)	0.00004221	<0.0001
**1940–1949 (33241 years of 558 persons) R^2^ = 0.03, P<0.001**				
sex (1:male, 0: female)	0.09513	(0.02952;0.16074)	0.03347	0.0045
age	0.2713	(0.03429;0.50831)	0,12092	0.0249
calendar year	0.00007559	(0.00002422;0.00012697)	0.00002621	0.0039
age*calendar year	−0.00013216	(−0.00025055;−0.00001378)	0.00006040	0.0287
**1960–1969 (5842 years of 152 persons) R^2^ = 0.02, P<0.001**				
sex (1:male, 0: female)	0.00886	(−0,06169;0.07941)	0.03599	0.8055
age	0.49659	(−0.07870;1.07188)	0.29346	0.0907
calendar year	−0.00000993	(−0.00006336;0.00004351)	0.00002726	0.7157
age*calendar year	−0.00024474	(−0.00053213;0.00004265)	0.00014660	0.0951

## Discussion

This paper is based on a large population-representative control sample of 2811 subjects. The data is estimated to be more detailed and complete than previous studies using aggregate data from health insurance funds [15], [16] or from a small number of hospitals or even a single selected hospital disregarding examinations outside the chosen hospital [17]. The big strength of our study is the livelong cohort approach which could consider simultaneously the temporal changes in diagnostic radiologic practice including technical advances in equipment and age and sex of the patient in a large sample, representative for the general population. To avoid any differential bias between cases and controls this analysis was restricted to controls. Additionally, data obtained from next to kin interviews were not considered, because their memories were likely less accurate than that of the persons themselves [14]. Nevertheless, a less than perfect recall of examinations is still possible especially by older subjects who's memories have to cover a large time period. However, Linet et al. compared interview data and medical records for previous medical conditions and surgery and did not find an association between agreement and age [18]. To minimize possible recall bias the standardized interviews were designed to evoke pertinent memories by a description of the typical setting of the examinations and pictures of the commonly used technical devices.

The study took place in Northern Germany. Systematic differences in radiologic practice between federal states would limit the generalizability of the observed results. Relevant differences, however, are likely restricted to the mass screening examinations which were regulated by federal law in Lower Saxony and Schleswig-Holstine but were optional in most other federal states of Germany, where participation may have been lower. Mass screening (originally for tuberculosis) was conducted biannually in the study area from 1948–1989 (Lower Saxony) and from 1947–1986 (Schleswig-Holstine) respectively.

In Figure 3 several peaks can be observed accompanying the first rise and decrease of the red bone marrow dose. This can be interpreted as an artifact of the quantification model, which assumed that the patient dose in conventional radiology is halved every ten years. While this has been proven true quite consistently overall since the 1940s [12], it can be expected that in real life the implementation of any one new technique was dispersed over a longer period of time causing a smoother temporal pattern in reality as is predicted by the model.

Mettler [19] and Brenner [20] reported a 10-fold increase of the frequency of radiologic examinations from 1950–2006 in the United States. This rise is confirmed by observations of other authors [21], [22] and is proven in this study for northern Germany in the time between approximately 1920–2000 as well. Additionally a steady increase with rising age can be detected that may correlate with the increasing morbidity and diagnostic demands with higher age.

While other authors describe merely an increase of dose over time [19], [20], this study shows a more complex dose development considering individual risk factors like age and sex. Thus in spite of the steady increase of frequency, the red bone marrow dose shows an increase till 1950 and a further one after 1980 with a drop between 1970 and 1980.

The first increase, almost exclusively caused by conventional and mass screening examinations, may be generated by the diffusion progress of x-ray equipment and the implementation of screening examinations due to the perceived spread of tuberculosis in the population. Around 1960, although the number of conventional examinations was still on a rise, technical improvement and advance in radiological practice reduced the average radiation dose per subject and year considerably. This may be related to improved surveillance and an increased awareness of possible radiation injury [23]. A sign for a sensitization towards the risk of radiation was the enactment of the first German Radiation Protection Ordinance Legislation (Strahlenschutzverordnung) in 1960. An additional but presumably less influential factor for the decreasing radiation dose could be the shift of the legal age for mass screening examinations from 2 to 14 (1961) and from 14 to 18 (1971), followed later by the phasing out of these compulsory examinations.

The second increase, caused by technically advanced examinations like computed tomography, contrast medium examinations, and cardiac catheter examinations, is linked to the implementation of CT in the 1980^s^. Additionally, cardiac catheter and contrast medium examinations became more common due to the development of amidotrizoat (gastrografin), a well tolerated oral contrast medium (1959), and the left heart cardiac catheter (1961) assuming some delay until common acceptance [24]. These examinations appear to be age-related, thus older subjects are exposed to doses caused by these kinds of examinations more often.

The accumulative dose of the red bone marrow decreases with more recent birth years. This is remarkable because at the same time the number of examinations rises considerably. Presumably, the reduction of patient dose by technical progress and improved radiological practice overcompensate the extra dose resulting from the ever increasing number of examinations at least after 1965–1975 (Table 2). Berrington et al. have estimated the cumulative risk of cancer to age of 75 years in both sexes from diagnostic x-ray and have calculated that diagnostic x-ray use in Germany causes 1.5% of the cumulative cancer risk, equivalent to a total of 2049 radiation-induced cases for both sexes (963 cases for men) per year [6]. The BEIR VII report describes a linear dose-response relationship between low levels of ionizing radiation (such as x-rays) and the development of solid cancers in humans [25]. Based on this report the estimated number of leukemia cases from exposure to 100 mSv would be 100 (confidence limits: 30–300) per 100,000 in males and 70 (confidence limits: 20–250) per 100,000 in females [25].

Females achieve lower doses than males in early birth years. A possible explanation for more examinations in young years could be that men adopt a more accident prone life style than women. In older ages a major reason might be the more common application of cardiac catheters to males.

### Conclusion

This study shows that sex and age of the subjects influenced the accumulated red bone marrow dose. In epidemiologic studies concerning the assessment of dose caused by medical diagnostic, the influence of sex and age can be sufficiently considered by the number and kind of examinations. In contrast, the influence of the calendar year should be taken into account separately. Thus the red bone marrow dose caused by medical diagnostic does not increase proportional with the increasing number of examinations, but depends highly on the technical standards and radiation protection survey of the respective calendar year. This could lead to a significant misestimating of the red marrow dose if considering only the number and kind of examinations.
